# Metabolic rate and resource depletion in the tick *Ixodes ricinus* in response to temperature

**DOI:** 10.1007/s10493-020-00568-1

**Published:** 2020-11-11

**Authors:** Saeed Alasmari, Richard Wall

**Affiliations:** School of Biological Sciences, University of Bristol, 24 Tyndall Avenue, Bristol, BS8 1TQ UK

**Keywords:** Blood-feeding, Climate change, Ixodes, Metabolite, Temperature threshold

## Abstract

Understanding the effects of temperature on the metabolic activity and the rate of depletion of energy reserves by *Ixodes ricinus* can represent an important contribution to explaining patterns of tick activity and the likely impacts of environmental change on tick and tick-borne disease risk. Here, a cohort of *I. ricinus* nymphs, males, and females was collected and placed into incubators at temperatures of between 5 and 30 °C. The protein, carbohydrate, total lipid, neutral lipid, and glycogen levels were measured for nymphs for up to 70 days and adults up to 42 days. In nymphs, at day 0, glycogen was the most abundant metabolite followed by carbohydrate, with relatively low concentrations of protein and lipids. For males, the concentrations of different metabolites were relatively similar. In contrast, for females, concentrations of glycogen and carbohydrate were relatively low compared to those of protein and neutral lipids. Significant exponential declines in metabolite concentrations of all metabolites were detected over time for all life-cycle stages and at all temperatures. Nymphs generally showed lower rates of resource depletion than adults at all temperatures. The lower thresholds for metabolic activity were estimated to be between −10 and −5 °C. The Q_10_ values, which describe the thermal sensitivity of metabolic rate, were estimated to be relatively low (1.5 for nymphs, 1.71 for males, and 1.63 for females) compared to insects where they are typically around 2.5 (range: 1.5–3), and this is considered to be an adaptation to increase survival during the extended inter-feed intervals.

## Introduction

Intermittent blood-feeding and digestion are key behavioural and physiological processes in hard ticks (Ixodidae) that are intimately associated with their role as vectors of pathogens (Randolph 2004). Unlike hematophagous insects, a blood-meal is taken only once by each life-cycle stage: the inter-feed interval is usually several months in univoltine species, ingestion is relatively slow, the volume of blood ingested is relatively large, and digestion is intracellular (Horn et al. 2009; Kongsuwan et al. 2010). Sufficient nutrition must be obtained at each meal and stored within the body to allow larvae and nymphs to undergo development to the next life-cycle stage and eventually initiate repeated questing until another host is located. For adults, accumulated metabolic reserves must also provide the resources for reproduction (Diehl et al. 1982). The longer ticks can survive between blood-meals, the higher the chance of encountering a new host; hence, the rate at which ticks use accumulated resources during the extensive off-host period is critical to their survival and reproductive success.

The primary immediate energy source for ticks is glucose, which is stored in the hydrated polymeric form of glycogen, but this is rapidly depleted (Moraes et al. 2007). Recent studies have shown that glycogen alone forms 39% of the mass of metabolites in *I. ricinus* nymphs compared to 15 and 10% in females and males, respectively (Alasmari and Wall 2020). Carbohydrate can be used to replenish glycogen reserves. Lipids also play key roles in tick metabolism, both as an energy source and structurally. Lipid is stored primarily the form of triglycerides in adipocytes, the main fat body cell, and additionally as cytoplasmic lipid droplets. Lipid metabolism is particularly associated with growth phases (Arrese and Soulages 2010). The measurement of lipid has been used previously to infer patterns of feeding within cohorts of *I. ricinus* derived from the field (Randolph et al. 2002; Abdullah et al. 2018). Proteins are essential for muscle development and the synthesis of cuticle, hormones and enzymes, synthesis of egg yolk and the production of sperm and gonadal proteins (Kongsuwan et al. 2010). Proteins may also be metabolised as a long-term energy reserve (Williams et al. 1986).

Patterns of metabolic activity are not uniform throughout life and increases are associated with feeding and moulting, followed by reduced levels of metabolic activity and energy utilisation for several weeks (Cuber et al. 2016). However, once energy levels have been depleted, the levels of activity and energy expenditure then increase as questing becomes more prolonged and increasingly persistent, and this makes ticks highly susceptible to environmental stresses such as dehydration (MacLeod 1935; Needham and Teel 1991; Bowman et al. 1996). The intermittent feeding of hard ticks is a relatively high-risk life-history strategy, and mortality rates in each life-cycle stage associated with questing must be counterbalanced, in terms of fitness, by the benefits from the reduced risk of injury gained by not spending long periods on an actively grooming host.

Strong non-linear relationships between temperature and many aspects of tick life-history have been demonstrated, although this relationship is not a simple one because saturation deficit will affect desiccation (Needham and Teel 1991) and temperature, in combination with day length, may affect various forms of behavioural or physiological diapause (Belozerov 2009). For example, researches on *Ixodes scapularis* have found that the power relationships observed between temperature and development rate in the laboratory could be used to predict dates for moulting, oviposition, and field-observed seasonal questing in larvae and nymphs, (Ogden et al. 2004; Burtis et al. 2019). However, other factors, such as the seasonal activity of questing adult ticks, were poorly predicted. An understanding of the pattern of metabolite use and depletion, particularly in responses to factors such as temperature, is important, not only to explain tick population dynamics and phenology, but also because it may contribute to a better understanding of the likely impacts of climatic changes on ticks, since this is likely to affect seasonal activity patterns and, consequently, may affect pathogen transmission (Gray et al. 2009; Burtis et al. 2016).

Energy budgets and factors affecting metabolic rate have been extensively studied in insects (Satake et al. 2000; Chown and Nicolson 2004) but are much less well understood in ticks. The study of metabolite concentrations and depletion has been greatly facilitated by the spectrophotometric techniques developed by van Handel (1985a, b), and recent studies have demonstrated that these methods can also be used to determine the entire energy budget of individual ticks (Alasmari and Wall 2020). The aim of the work described here was therefore to investigate the effects of temperature on the rate of depletion of metabolic reserves over time in nymphs and adults of *I. ricinus* collected from the field.

## Materials and methods

### Tick collection and environmental conditions

A total of 2160 ticks were collected from Ashton Court Park near Bristol on a single day (day −14) in the middle of March 2019. The samples collected were temporarily stored at 4 °C for 6 h before being divided into groups based on life-cycle stage. Nymphs, adult males, and adult females of *I. ricinus* were then divided into six groups of 100–150 individuals, which were transferred into 1.2-l plastic buckets with tightly fitting lids (12.5 × 18 cm). Each bucket contained 3 kg of dampened horticultural silver sand (Melcourt, Tetbury, UK) to raise the humidity experienced by the ticks to > 80%; humidity was not controlled further, but conditions were similar for all groups and humidity was sufficiently high to allow high survival at all temperatures. All buckets were placed in incubators (Sanyo, MLR-351) set at temperatures of 5, 10, 15, 20, 25, and 30 °C, and kept under diel periods of L8:D16 h. A relatively short photoperiod was used to reflect the time of year when the ticks were collected, as a change from short to long daylight could influence physiological changes associated with diapause (Gray et al. 2016). Ticks were initially maintained under these conditions for 2 weeks before the experiment was considered to have started (day 0) to allow them to adapt to their new laboratory environment. For nymphs, the concentrations of protein, carbohydrate, total lipid, neutral lipid, and glycogen were measured at days 0, 14, 28, 42, 56, and 70. For the adult males and females, the measurements of the same metabolites were performed on days 0, 14, 28, and 42 only.

### Biochemical measurements

The spectrophotometric methods used were as described in Alasmari and Wall (2020) based on those developed by van Handel (1985a, b). In brief, for the protein analysis, the ticks were first individually placed in a sterilised borosilicate tube (12 ml) and crushed with a clean glass rod. Next, 1500 µl of a phosphate buffer solution [100 mM of monopotassium phosphate, 1 mM of ethylenediaminetetraacetic acid, and 1 mM of dithiothreitol (pH 7.4)] was added to extract the protein component. Thereafter, the homogenate was placed on ice (for about 1 min) prior to processing. For the nymphs, 1000 µl of the homogenate was transferred to a cuvette and mixed with 1000 µl of Bradford reagent (Sigma, Dorset, UK). For the adult males and females, because the sample size was smaller, 50 µl of the homogenate was transferred to a cuvette and mixed with 1500 µl of Bradford reagent. Individual cuvettes were incubated at room temperature for 5 min, after which the absorbance was immediately measured at a wavelength of 595 nm using a spectrophotometer (Biochrom Biowave II, Cambridge, UK).

For the analysis of the other metabolites, a single individual tick was placed in a clean borosilicate tube and crushed, as described above. To dissolve water-soluble carbohydrates and the total fats, 200 µl of 2% sodium sulphate solution (VWR International, Leicestershire, UK) and 1500 µl of a chloroform/methanol mixture (1:2, vol/vol) were added to each tube. This solution was transferred into a 2-ml Eppendorf tube and centrifuged (Eppendorf Centrifuge 5418R; Lutterworth, UK) for 15 min at 180×*g* at 4 °C. For the analysis of the total lipids, neutral lipids and carbohydrates, the supernatant was transferred to a new tube.

For the carbohydrate analysis, 200 µl of the supernatant from individual samples was transferred to a borosilicate tube and placed in a water bath at 90 °C for 40 s so that the solvent would evaporate; this was done until about 20 µl was left. Thereafter, 1 ml of freshly prepared anthrone reagent (Sigma) at a concentration of 1.42 g/l of 70% sulphuric acid (VWR International) was added to each sample and incubated for 15 min at 25 °C. Subsequently, the tubes were heated for 15 min at 90 °C and cooled at room temperature for 15 min. Then, the samples were placed in a microcuvette and read in a spectrophotometer at an absorbance wavelength of 625 nm.

For the glycogen analysis, the pellets were washed twice with 400 µl of 80% methanol to remove sodium sulphate. Then, vigorous vortexing was performed at full speed for up to 20 s; this was followed by centrifugation for 5 min at 180×*g* and 4 °C. Once the supernatant was removed, 1 ml of fresh anthrone reagent was added, and the mixture was incubated at 90 °C for 15 min. The samples were cooled on ice to stop the reaction and filtered through low-protein-binding membranes with a pore diameter of 0.45 µm (Fisher Scientific, Leicestershire, UK). Finally, the absorbance was read with a spectrophotometer at a wavelength of 625 nm.

The total lipids were quantified using a vanillin assay. For this assay, 200 µl of the supernatant was added into a new borosilicate tube that was placed in a heating block at 90 °C until total evaporation was achieved. Then, 40 µl of 95% sulphuric acid (VWR International) was added to the residue in the tube, heated at 90 °C for 2 min, and cooled on ice. Next, 960 µl of the freshly prepared 1.2 g/l vanillin reagent (Fisher Scientific) in 68% phosphoric acid (Sigma) was added, and the solution was incubated at room temperature for 15 min. Absorbance was read with a spectrophotometer set to a wavelength of 525 nm.

The neutral lipid content was measured by placing 500 µl of the supernatant in a new tube that was heated at 90 °C; this was done until the solvent had completely evaporated. Next, 1 ml of chloroform was added, and the solution was transferred to a new tube. Next, the fats were resolubilised by adding 200 mg of dry silicic acid (Sigma) to each sample and mixing. The mixed solution was then centrifuged at 180×*g* at 4 °C for 10 min to remove any polar lipids present in the silicic acid. From the final supernatant, 200 µl was transferred to a new tube using a pipette. The procedure above was repeated, and the absorbance was read with a spectrophotometer set to a wavelength of 525 nm. For each temperature condition, extractions were performed on 12 nymphs, 12 adult males, and 12 adult females.

### Standard curves

Standard curves of the absorbance of known metabolite concentrations were created so that the measured spectrophotometric values could be used to determine the corresponding metabolite concentrations. Care was taken to ensure that the relationship between the concentrations and the spectrophotometric values was linear and that the curves covered the entire range of the measured concentrations (from below the lowest value to the highest value). For determining the protein concentration, a standard curve was generated with a dilution series of bovine serum albumin (1 mg/ml; Sigma) that was treated as described above. For determining the carbohydrate and glycogen concentrations, a standard curve was generated using glucose (1 mg/ml; Sigma) at a range of dilutions. For the lipid analysis, a standard curve was generated using glycerol trioleate (1 mg/ml; Sigma) at different dilutions. Five independent repeats of different serial dilutions were conducted to produce each standard curve.

### Data analysis

Analyses were performed with the R-Studio statistical package v.3.5.3 (R Foundation for Statistical Computing, Vienna, Austria) and SPSS v.26 (IBM SPSS Statistics, Armonk, NY, USA). The relationships between metabolite concentration and time and the rate of metabolite depletion and temperature were determined by best-fit regression analysis and differences between metabolite concentrations by one- and two-way ANOVA with Tukey post-hoc tests.

## Results

### Initial metabolite concentrations

**Fig. 1:**
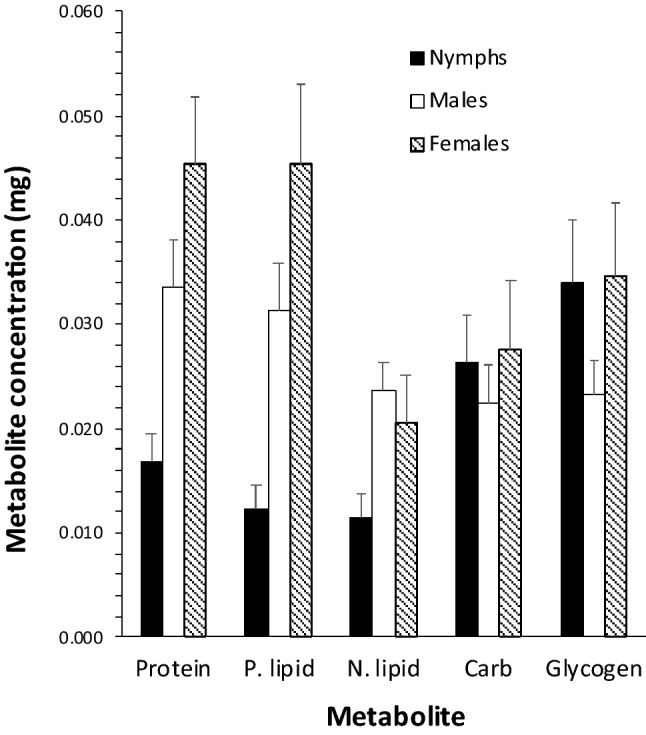
Mean (± SD) concentrations of protein, phospholipid (p. lipid), neutral lipid (n. lipid), carbohydrate (carb) and glycogen at day 0, found in nymphal, male, and female *Ixodes ricinus* ticks collected by blanket dragging in March, 2019

At day 0, there were highly significant differences in the concentrations of metabolites present (F_4,1074_ = 262.87, P < 0.001) for all life cycle stages (F_2,1074_ = 791.7, P < 0.001) with a significant interaction between metabolite and life cycle stage (F_8,1074_ = 227.9, P < 0.001; Fig. 1). In nymphs, glycogen was the most abundant metabolite, followed by carbohydrate, with relatively low concentrations of protein and lipid (F_4,__355_ = 459.0, P < 0.001), with no significant difference between the concentrations of neutral lipid and phospholipid. For males, the concentrations of metabolites were relatively more even, with no significant difference between the concentrations of neutral lipid, carbohydrate, and glycogen, which were significantly lower than the concentrations of phospholipid and protein (F_4,360_ = 132.9, P < 0.001; Fig. 1. In contrast, for females, relatively high concentrations of protein and neutral lipid were present at concentrations that were not significantly different from each other but were higher than other metabolites, which were all different from each other (F_4,360_ = 202.8, P < 0.001; Fig. 1).

### Metabolite depletion over time

Significant exponential declines in metabolite concentrations were detected over time for all life-cycle stages and at all temperatures (Tables 1, 2 and 3). The slopes of the regressions indicate that nymphs generally show lower rates of resource depletion than adults at all temperatures. The overall mean (± SD) rate of depletion was 0.016 ± 0.0058 mg/day for nymphs, 0.019 ± 0.0085 for males, and 0.023 ± 0.0088 for females. Over a period of 42 days at 5 °C, nymphs lost an average of 25% of their initial metabolite mass. In contrast, at the same temperature and over the same time period, adults showed losses of on average 35% for males and 40% for females. At 30 °C nymphs lost an average of 55% of their initial metabolite mass over 42 days, whereas adults lost an average of 73 and 78% for males and females, respectively. Extrapolation from these results suggests that starvation would occur at between 50 and 70 days at 25–30 °C and 100–200 days at 5 °C.

**Table 1:** Relationships between concentration for each metabolic component and time (days) for *Ixodes ricinus* (nymphs) that were maintained at temperatures of 5 to 30 °C, with the equation of the best fit negative exponential line, R^2^, F, and significance of the regression (P)

Metabolic components	Temperature (°C)	Regression			
		Equation	R^2^	F	P
Protein	5	y = 0.0179e^−0.008x^	0.62	118	0.0001
	10	y = 0.0171e^−0.009x^	0.59	101.3	0.0001
	15	y = 0.0165e^−0.010x^	0.71	178.7	0.0001
	20	y = 0.0168e^−0.013x^	0.74	208.6	0.0001
	25	y = 0.0161e^−0.017x^	0.81	304.4	0.0001
	30	y = 0.0176e^−0.023x^	0.83	348.4	0.0001
Phospholipid	5	y = 0.013e^−0.008x^	0.47	63.3	0.0001
	10	y = 0.0125e^−0.009x^	0.58	96.86	0.0001
	15	y = 0.0126e^−0.011x^	0.74	205.7	0.0001
	20	y = 0.0122e^−0.015x^	0.72	181.5	0.0001
	25	y = 0.0125e^−0.018x^	0.82	326.1	0.0001
	30	y = 0.0125e^−0.022x^	0.78	254.9	0.0001
Neutral lipid	5	y = 0.0126e^−0.012x^	0.64	124.5	0.0001
	10	y = 0.0128e^−0.014x^	0.66	140.2	0.0001
	15	y = 0.0126e^−0.017x^	0.75	212.2	0.0001
	20	y = 0.0128e^−0.021x^	0.74	208.8	0.0001
	25	y = 0.0118e^−0.023x^	0.84	378.8	0.0001
	30	y = 0.0123e^−0.028x^	0.85	399.3	0.0001
Carbohydrate	5	y = 0.0283e^−0.007x^	0.46	61.33	0.0001
	10	y = 0.0294e^−0.011x^	0.65	131.3	0.0001
	15	y = 0.0289e^−0.013x^	0.64	127.5	0.0001
	20	y = 0.0291e^−0.015x^	0.70	169.2	0.0001
	25	y = 0.0295e^−0.018x^	0.76	223.8	0.0001
	30	y = 0.0327e^−0.024x^	0.80	286.9	0.0001
Glycogen	5	y = 0.0336e^−0.009x^	0.58	100.6	0.0001
	10	y = 0.035e^−0.013x^	0.73	193	0.0001
	15	y = 0.0342e^−0.015x^	0.74	206.4	0.0001
	20	y = 0.0352e^−0.017x^	0.83	349.7	0.0001
	25	y = 0.0353e^−0.02x^	0.83	345.3	0.0001
	30	y = 0.0386e^−0.027x^	0.83	360.7	0.0001

**Table 2:** Relationships between concentration for each metabolic component and time (days) for *Ixodes ricinus* (males) that were maintained at temperatures of 5 to 30 °C, with the equation of the best fit negative exponential line, R^2^, F, and significance of the regression (P)

Metabolic components	Temperature (°C)	Regression			
		Equation	R^2^	F	P
Protein	5	y = 0.0337e^−0.006x^	0.24	14.81	0.0003
	10	y = 0.0335e^−0.009x^	0.50	46.52	0.0001
	15	y = 0.0342e^−0.013x^	0.65	87.07	0.0001
	20	y = 0.0329e^−0.016x^	0.70	107	0.0001
	25	y = 0.0317e^−0.022x^	0.78	167.1	0.0001
	30	y = 0.0308e^−0.028x^	0.76	146.3	0.0001
Phospholipid	5	y = 0.0334e^−0.008x^	0.37	28.02	0.0001
	10	y = 0.0323e^−0.012x^	0.56	58.95	0.0001
	15	y = 0.0315e^−0.017x^	0.64	82.44	0.0001
	20	y = 0.03e^−0.023x^	0.80	190.6	0.0001
	25	y = 0.0288e^−0.029x^	0.75	139.4	0.0001
	30	y = 0.0289e^−0.033x^	0.84	249.8	0.0001
Neutral lipid	5	y = 0.0238e^−0.009x^	0.33	23.09	0.0001
	10	y = 0.0233e^−0.013x^	0.48	42.83	0.0001
	15	y = 0.0231e^−0.016x^	0.66	89.92	0.0001
	20	y = 0.0213e^−0.02x^	0.61	72.78	0.0001
	25	y = 0.0206e^−0.025x^	0.64	84.72	0.0001
	30	y = 0.0207e^−0.035x^	0.79	174.6	0.0001
Carbohydrate	5	y = 0.0224e^−0.012x^	0.48	42.81	0.0001
	10	y = 0.023e^−0.017x^	0.65	85.65	0.0001
	15	y = 0.0227e^−0.021x^	0.73	129.3	0.0001
	20	y = 0.0221e^−0.025x^	0.73	126.5	0.0001
	25	y = 0.0226e^−0.030x^	0.80	193.7	0.0001
	30	y = 0.0218e^−0.033x^	0.80	194.1	0.0001
Glycogen	5	y = 0.0239e^−0.008x^	0.24	15.21	0.0003
	10	y = 0.0228e^−0.008x^	0.39	29.47	0.0001
	15	y = 0.0229e^−0.012x^	0.40	31.32	0.0001
	20	y = 0.0231e^−0.014x^	0.43	35.19	0.0001
	25	y = 0.0225e^−0.018x^	0.61	75.03	0.0001
	30	y = 0.0223e^−0.024x^	0.74	131.2	0.0001

**Table 3:** Relationships between concentration for each metabolic component and time (days) for *Ixodes ricinus* (females) that were maintained at temperatures of 5 to 30 °C, with the equation of the best fit negative exponential line, R^2^, F, and significance of the regression (P)

Metabolic components	Temperature (°C)	Regression			
		Equation	R^2^	F	P
Protein	5	y = 0.0456e^−0.011x^	0.47	41.98	0.0001
	10	y = 0.0476e^−0.016x^	0.56	59.39	0.0001
	15	y = 0.0488e^−0.021x^	0.70	110.4	0.0001
	20	y = 0.0497e^−0.025x^	0.80	187.1	0.0001
	25	y = 0.05e^−0.031x^	0.79	181.7	0.0001
	30	y = 0.0507e^−0.036x^	0.84	259.5	0.0001
Phospholipid	5	y = 0.0467e^−0.011x^	0.56	59.16	0.0001
	10	y = 0.0461e^−0.016x^	0.61	72.32	0.0001
	15	y = 0.0469e^−0.021x^	0.69	105.1	0.0001
	20	y = 0.0463e^−0.024x^	0.74	132.8	0.0001
	25	y = 0.0458e^−0.031x^	0.78	170.6	0.0001
	30	y = 0.0482e^−0.036x^	0.87	309.8	0.0001
Neutral lipid	5	y = 0.0205e^−0.014x^	0.50	47.14	0.0001
	10	y = 0.0205e^−0.018x^	0.56	60.57	0.0001
	15	y = 0.0201e^−0.021x^	0.61	73.19	0.0001
	20	y = 0.0206e^−0.029x^	0.68	100.8	0.0001
	25	y = 0.0206e^−0.033x^	0.75	140.2	0.0001
	30	y = 0.0202e^−0.042x^	0.80	191.7	0.0001
Carbohydrate	5	y = 0.0262e^−0.011x^	0.28	18.08	0.0001
	10	y = 0.0267e^−0.014x^	0.45	38.41	0.0001
	15	y = 0.0275e^−0.018x^	0.59	66.3	0.0001
	20	y = 0.0276e^−0.021x^	0.56	60.73	0.0001
	25	y = 0.0279e^−0.026x^	0.72	119.5	0.0001
	30	y = 0.0275e^−0.034x^	0.76	146.7	0.0001
Glycogen	5	y = 0.0362e^−0.013x^	0.49	44.39	0.0001
	10	y = 0.0356e^−0.014x^	0.57	62.8	0.0001
	15	y = 0.0344e^−0.016x^	0.60	70.12	0.0001
	20	y = 0.0333e^−0.021x^	0.58	64.28	0.0001
	25	y = 0.0337e^−0.031x^	0.69	105.8	0.0001
	30	y = 0.0317e^−0.033x^	0.74	135	0.0001

### Metabolite depletion with temperature

**Fig. 2:**
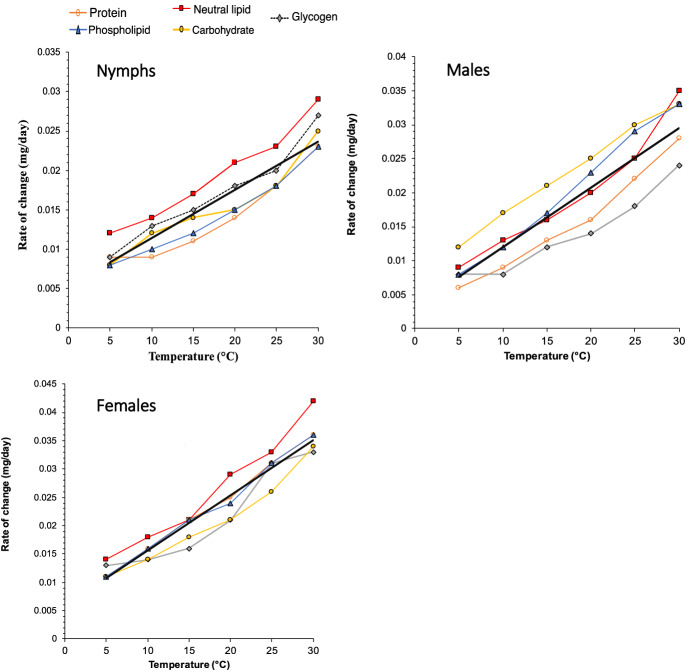
Rate of change of various metabolites over time in *Ixodes ricinus* nymphs, males, and females at 5–30 °C. Points are joined for clarity. The solid black lines indicate the best fit linear regression fitted to all data (nymphs: Y = 0.0006 X + 0.0053, R^2^ = 0.834; males: Y = 0.0009 X + 0.0033, R^2^ = 0.804; females: Y = 0.001 X + 0.0059, R^2^ = 0.916, all P < 0.001)

The slopes of the relationships between metabolite concentration and time describe the rate of loss of each class of compound (mg/day). A plot of the slope of the relationship between metabolite concentration and time against temperature therefore describes the relationship between the rate of loss of each compound class and temperature (Fig. 2). Consistent patterns in the rate of use of particular metabolites were evident between different life-cycle stages. For nymphs, the rates of use of neutral lipid and glycogen were consistently high and the rate of loss of protein was low, particularly at low temperatures. In contrast, for males, carbohydrate concentrations declined most rapidly and glycogen the least. Finally, for females, there was much less variation in the rate of metabolite use compared to males or nymphs but a consistent pattern emerged, with lipid concentrations declining at a higher rate than other metabolites. The Q_10_ values, determined from a linear regression fitted to all the metabolite data (Fig. 2), gave values of 1.5 for nymphs, 1.71 for males, and 1.63 for females. Extrapolation of this linear regression indicates that the lower temperature threshold for metabolic activity in *I. ricinus* for is − 8.8 °C for nymphs, − 4.1 °C for males, and − 5.9 °C for females.

## Discussion

Metabolic rate and changes in the rates of metabolite use are associated with extrinsic factors such as temperature and humidity and intrinsic factors associated with feeding, moulting, and reproduction. Understanding how these factors influence changes in metabolic rate and resource depletion are important for explaining patterns of tick phenology in the field and for prediction of changes in distribution and abundance that may occur in response to environmental change (Hancock et al. 2011).

The ticks used for the present study were collected by blanket dragging in March 2019. At this time of year, questing activity of nymphs and adults in southwest England would be expected to be at its peak. Populations of nymphs and adults would largely be composed of cohorts which fed the previous year and either moulted the previous autumn or which overwintered and moulted in early spring prior to collection, depending on precisely when they had fed. Amongst the ticks collected, at experimental day 0, clear differences in metabolite concentrations between life cycle stages were apparent: these were largely associated with the differences in the relative amounts of protein compared to glycogen. In nymphs, glycogen represented 34% of the total mass of metabolites measures, followed by carbohydrate at 26%, whereas protein represented only 16% of the mass of metabolites present in nymphs. In contrast, glycogen composed only 12% of the mass of metabolites in females and 18% in males, whereas protein represented 26% of the mass in females and 25% in males. These differences are consistent with those recorded previously (Alasmari and Wall 2020). The relatively low glycogen and carbohydrate levels in adults may be associated with the fact that the adult population is composed predominantly of individuals that fed as nymphs early the previous year and moulted the previous autumn and so by March are relatively resource depleted (Randolph et al. 2002; Abdullah et al. 2018). In contrast, the nymphs may be derived from larvae that fed later in the previous year and may have moulted in late autumn or early spring and so have relatively higher energy reserves and particularly are richer in glycogen and carbohydrate. This hypothesis could be tested by examining the full metabolite profile of adults collected at different times, particularly in late autumn. If this is correct, over time, because nymphs were relatively better resourced with higher initial short-term energy resources, their lipid and glycogen rates of depletion would be higher than those of other metabolites and their rate of protein loss would be low. In contrast to nymphs, for adults, males were depleting carbohydrate and lipid at the highest rates, whereas in females the highest rates of depletion were seen in lipid and protein concentrations; in adults, the rates of glycogen depletion were relatively low because their initial glycogen concentrations available at day 0 were also relatively low. The rate of depletion of each metabolite during the trial was relatively constant in response to temperature and was best described by an exponential model. No major changes in the rank order of the rates of metabolite depletion over time in response to changes in temperature could be detected, although this might have been predicted in nymphs as they depleted their glycogen and carbohydrate reserves. Examination of a full season-long metabolic profile in tick cohorts would be a useful next step.

In a study that considered only stored lipids, the lipid reserves of field-collected *I. ricinus* nymphs, collected in early summer, were estimated to be sufficient to allow survival without feeding for up to 100 to 250 days at 15 °C, depending on whether they had fed as larvae the previous autumn or that year, respectively (Abdullah et al. 2018). Here, the results suggest that, amongst the cohort collected, complete resource depletion would occur at 45–70 days at 25–30 °C and 200 days at 5 °C, assuming humidity was sufficiently high not to result in desiccation. This fits with previous estimates from observations in the field (Steele and Randolph 1985) and arenas (Randolph and Storey 1999), which indicated a maximum questing period of about 120 days for nymphs.

The thermal sensitivity of metabolic rate, often described by an organism’s Q_10_, is the magnitude of change in metabolic rate for a 10 °C change in temperature. In insects, Q_10_ values range from 1.5 to 3, with a mode of 2.5 (Woods et al. 2003). Here, Q_10_ values were 1.5 for nymphs, 1.71 for males and 1.63 for females. These values are relatively low, and this reflects the low metabolic rate that helps to increase survival during the extended inter-feed intervals. It has previously been suggested that ixodid ticks have a metabolic rate which is typically 13% below that of most arthropods (Lighton and Fielden 1995). Why the metabolic rate of nymphs was lower than those of adults is unclear, but it may be that metabolic rate increases in response to the degree of resource depletion, which leads to higher levels of questing activity.

Temperature is considered the most important factor influencing the initiation of *I. ricinus.* For questing, a maximum daily air temperature of 7–8 °C has been proposed as necessary (MacLeod,1935). In constant-temperature laboratory trials, the minimum threshold temperature for metabolic activity was estimated to be 5.7 °C for populations derived from Scotland, 7.9 °C for Wales, 7.0 °C for England, 9.3 °C for France at low altitude, and 6.9 °C for France at high altitude (Tomkins et al. 2014; Perret et al. 2000) found that questing ticks were always collected when the temperature reached or exceeded 5.2 °C. Environmental conditions with relative humidity higher than 45% and an ambient temperature exceeding 2.5 °C were considered to be required for questing by *I. ricinus* (Hubalek et al. 2003). Some element of genetic variation reflecting local adaptation is also evident (Gilbert et al. 2014). Here, however, assuming that a linear extrapolation from the observed temperature range is appropriate, the data presented suggest that the lower threshold for metabolic activity is between − 10 and − 5 °C, which is probably close to the lower lethal temperature of this species. An understanding of the lower lethal temperatures and the fact that that metabolic activity is likely to be occurring even at temperatures below the threshold for questing may help to refine attempts to model tick phenology using climate simulations, as it helps to define the climate envelope within which this species can survive. Further work explicitly examining tick metabolism at low temperatures would therefore be of value. Interestingly, complex relationships between metabolite concentrations and pathogen infection and transmission may also occur (Herrmann et al. 2013) and require more detailed consideration in tick-borne disease models.
